# Family support mediates the relationship between medication adherence and quality of life among patients with hypertension in Ghana: A cross-sectional study

**DOI:** 10.1371/journal.pone.0351632

**Published:** 2026-06-24

**Authors:** Cynthia Serwaa Akoto Osei, Jacob Solomon Idan, Edmund Baah-Akyeamfour, Bhavana Singh, Daniel Boateng

**Affiliations:** 1 Department of Epidemiology and Biostatistics, School of Public Health, Kwame Nkrumah University of Science and Technology, Kumasi, Ghana; 2 University Hospital, Kwame Nkrumah University of Science and Technology, Kumasi, Ghana; University of Energy and Natural Resources, GHANA

## Abstract

**Background:**

Hypertension poses significant challenges to quality of life globally. While medication adherence is essential for managing hypertension, psychosocial factors like family support may influence both adherence and quality of life. This study investigated the mediating role of family support in the relationship between medication adherence and quality of life among persons with hypertension in Ghana.

**Methods:**

A cross-sectional study was conducted among 365 patients with hypertension attending Kwame Nkrumah University of Science and Technology Hospital in Kumasi, Ghana. Data were collected using structured questionnaires, including the Short Form-36 for quality of life assessment, the Family Adaptation, Partnership, Growth, Affection, Resolve scale for family support measurement, and the Morisky Medication Adherence Scale. Mediation analysis was performed using R statistical software.

**Results:**

Five models with increasing levels of adjustment were constructed for the mediation analysis. Higher medication adherence was significantly associated with better quality of life (Total Effect: (β) = 1.07, 95% CI: 0.63–1.50, p < 0.001). Family support significantly mediated this relationship (Average Causal Mediation Effect = 0.09, 95% CI: 0.02–0.19, p = 0.008), accounting for 8% of the total effect. The direct effect of medication adherence on quality of life remained significant after adjusting for family support (Average Direct Effect = 0.98, 95% CI: 0.52–1.42, p < 0.001). The mediation effect persisted after adjusting for socio-demographic characteristics, knowledge score, healthcare access, and lifestyle factors (mediated proportion: 8.9%, p = 0.038). Patients from highly functional families demonstrated better quality of life with a unit increase in medication adherence score.

**Conclusion:**

Family support partially mediates the relationship between medication adherence and quality of life of persons with hypertension. Interventions to improve medication adherence and quality of life should incorporate strategies to enhance family support systems. Healthcare providers should consider the complex family dynamics when developing comprehensive management plans for persons with hypertension.

## Introduction

Hypertension is a major public health challenge globally with prevalence of 34.1%, particularly in low- and middle-income countries (LMICs) and Sub-Saharan Africa (SSA) [1–3]. The African regional prevalence is estimated at 27% [4]. In Ghana, the prevalence of hypertension is estimated at 34% across different regions, with the elderly populations experiencing higher rates [4,5]. The prevalence is estimated at 37.4% in Ashanti region [2]. Hypertension significantly affects patients’ quality of life (QoL), encompassing physical, psychological, and social well-being [2,6]. Medication adherence, defined as the extent to which patients take medications as prescribed by healthcare providers, is crucial for effective hypertension management and quality of life [7]. Poor adherence to antihypertensive medications is associated with uncontrolled blood pressure, increased risk of complications (heart disease, cerebrovascular accidents, kidney disease), and reduced QoL [8]. While pharmacological treatments for hypertension are available, their therapeutic benefits are linked to patient adherence behaviors [8].

The biopsychosocial model of health recognizes that biological, psychological, and social factors interact to influence health outcomes [9]. Within this framework, family support represents an important social determinant that may influence both medication adherence and QoL in hypertension management [10]. Family support encompasses emotional, instrumental, informational, and appraisal assistance provided by family members, which may facilitate adherence behaviors and enhance psychological well-being [9,10].

While some studies have examined the burden [2,5], direct relationships between medication adherence and blood pressure control among hypertensives who are enrolled on the National Health Insurance Scheme [11], none have investigated the potential mediating role of family support in the relationship between medication adherence and QoL, particularly in the Ghanaian context. This study aimed to investigate whether family support meaningfully mediates the relationship between medication adherence and QoL among persons with hypertension attending Kwame Nkrumah University of Science and Technology (KNUST) Hospital in Kumasi, Ghana. The findings of this study could inform the development of comprehensive interventions that leverage family resources to improve medication adherence and QoL among hypertensives.

## Methods

### Study design and setting

This cross-sectional study was conducted at the Outpatient Department (OPD) of KNUST Hospital in Kumasi, Ghana, between January and March 2025. KNUST Hospital is a primary healthcare facility that serves approximately 200,000 people annually, including university staff, students, and surrounding communities. The hospital has specialized clinics for chronic disease management, including a dedicated hypertension clinic that operates twice weekly [12].

### Study participants

Participants included in this study were persons who had been diagnosed with hypertension for at least six months and were receiving treatment at the hypertension clinic of KNUST Hospital. Inclusion criteria for recuitment were: (1) confirmed diagnosis of hypertension for at least six months, (2) age ≥ 18 years, (3) current use of antihypertensive medication(s), and (4) ability to provide informed consent. Patients with severe cognitive impairment, psychiatric disorders, or communication difficulties were excluded from the study.

### Sample size determination

The sample size was determined using the Cochrane formula for a finite population, considering an annual general OPD attendance of approximately 4,000. Based on previous study in Ashanti Region [2], the estimated prevalence of hypertension was 37%. Using a precision level of 5%, the initial calculated sample size was 332. After adjusting for an anticipated non-response rate of 10% that may be linked to disinterest or fatigue during engagement at the point of care, the minimum required sample size increased to 365 participants.

### Study sampling

Systematic random sampling was employed to recruit participants from the hypertension clinic of KNUST Hospital. The hypertensive clinic register listed 957 patients, and with a target sample size of 365, a sampling interval (k) of 3 was determined (957/365 ≈ 2.6, rounded to 3). A random starting point between 1 and 3 was selected, after which every third patient attending the clinic during the study period was approached for participation. Recruitment continued using this procedure until the target sample size was achieved.

### Data collection tool and procedure

Data was collected using a structured questionnaire developed based on relevant literature and input from experts in hypertension management, epidemiology, and health psychology. It was pretested among 20 patients with hypertension at a different healthcare facility to ensure clarity, relevance, and cultural appropriateness. Data collection took place in a private room at the OPD to ensure confidentiality. Trained research assistants conducted face-to-face quantitative survey using an electronic version of a structured questionnaires. Quantitative survey with study participants lasted approximately 30–45 minutes. The assistants also performed anthropometric measurements and reviewed medical records for relevant clinical information under the supervision of healthcare providers.

### Study variables

Data was collected on socio-demographic characteristics (age, gender, educational level, and occupation) and clinical measurements (height and weight for Body Mass Index (BMI) calculation and blood pressure measurements using calibrated automated devices). In addition, quality of life was assessed across eight domains, including physical functioning, role limitations due to physical health, bodily pain, general health perceptions, vitality, social functioning, role limitations due to emotional problems, and mental health using the Short Form-36 (SF-36) questionnaire [13]. Scores were transformed to a 0–100 scale, with higher scores indicating better QoL. The overall QoL score was calculated as the mean of all domain scores. An 8-item Morisky Medication Adherence Scale (MMAS) was utilised to assess adherence to antihypertensive medications [14]. Scores range from 0 to 8, with higher scores indicating better adherence. Adherence was categorised as low (<6), medium (6 to <8), or high (8). Permission to use the scale was obtained through the acquisition of a formal license from the Morisky Medication Adherence Scale developers. Family Support was assessed with a 5-item Family Adaptation, Partnership, Growth, Affection, Resolve (APGAR) scale to measure perceived family support across five dimensions: Adaptation, Partnership, Growth, Affection, and Resolve [15]. Scores range from 0 to 10, with higher scores indicating better family functioning. Family functioning was categorised as severely dysfunctional (0–3), moderately dysfunctional (4–6), or highly functional (7–10). The questionnaire also assessed patients’ knowledge of hypertension, including causes, symptoms, complications, and management. Further questions were asked to assess ease of healthcare access and satisfaction with healthcare service quality. Additionally, information on dietary habits and physical activity was collected.

### Data management

Completed questionnaires were thoroughly reviewed by the research team to ensure completeness and internal consistency before acceptance. This process involved systematic checks for inconsistencies, entry errors, missing values, and outliers. Logical checks and validation rules were applied to detect and correct anomalies. The final dataset was confirmed to be complete, with no missing values observed, ensuring data quality and reliability for subsequent analysis.

### Data analysis

Data were analyzed using R version 4.2.0 [16]. Descriptive statistics summarised participant characteristics, with continuous variables presented as means (±SD) and categorical variables as frequencies and percentages. Pearson’s correlation coefficient assessed bivariate associations between medication adherence, family support, and QoL. Mediation analysis using the approach described by Baron and Kenny [17] and implemented in the ‘mediation’ package in R examined whether family support mediated the relationship between medication adherence and QoL. Two main regression models were specified for the mediation analysis. The first, mediator model, examined the association between medication adherence and family APGAR scores. The second, outcome model, assessed the relationship between QoL and both medication adherence and family APGAR scores.

Five models with increasing levels of adjustment were constructed for each main model. The initial model was an unadjusted model. The second model included adjustments for sociodemographic factors such as age, gender, and education. The third model further adjusted for participants’ knowledge scores on hypertension. The fourth model incorporated additional adjustments for healthcare access and satisfaction, while the fifth and final model also included lifestyle factors such as dietary habits and physical activity. Each of these models comprised two components: a mediator model that assessed the relationship of family support with medication adherence and relevant covariates, and an outcome model that assessed the relationship of QoL with medication adherence, family support, and covariates. The analysis estimated the average causal mediation effect (ACME), average direct effect (ADE), total effect, and the proportion of the effect that was mediated. These estimates were obtained through bootstrapping with 1,000 simulations to generate 95% confidence intervals. The mediation analysis was applied to the physical and mental component summaries of QoL scores. Statistical significance was determined at a threshold of p < 0.05.

### Ethical considerations

The study adhered strictly to the Declaration of Helsinki by obtaining ethical approval from the Committee on Human Research, Publication and Ethics of KNUST School of Medicine and Dentistry (CHRPE/AP/478/24). Prior to data collection, research procedures were thoroughly explained to participants, including potential risks and benefits, study duration, rights to participation or withdrawal, and assurance of confidentiality and anonymity. All participants provided written informed consent. Participants were informed of their right to withdraw from the study at any time without penalty or impact on their medical care.

## Results

### Sociodemographic and clinical characteristics of participants

A total of 365 participated in the study, representing a response rate of 100%. The socio-demographic and clinical characteristics of the 365 patients with hypertension in this study (see Table 1) revealed a mean age of 54.8 (±15.2) years, with 37.0% being elderly (>60 years). Males constituted 50.4% of the study participants. Regarding education, 34.2% had tertiary education, while 19.2% had only primary education. The mean BMI was 28.7 (±5.9) kg/m², with 35.5% of participants being obese. The mean current systolic and diastolic blood pressure were 135.1 (±9.1) mmHg and 85.5 (±11.8) mmHg, respectively. The mean Quality of Life (QoL) score was 63.1 (±6.6). The mean medication adherence score was 5.0 (±1.6), and the mean family support score was 6.7 (±2.2). The majority of participants (69.6%) reported easy access to healthcare, though only 36.1% were satisfied with healthcare quality. Most participants (84.7%) engaged in frequent physical activity, and 70.4% reported fair dietary habits.

**Table 1:** Sociodemographic and clinical characteristics of persons with hypertension.

Characteristic	Frequency (%) (N = 365)
Age (years)*	54.8 (15.2)
**Age group**	
Young Adult [<40 Years]	61 (16.7%)
Adult [40–60 Years]	169 (46.3%)
Elderly [Above 60 Years]	135 (37.0%)
**Gender**	
Female	181 (49.6%)
Male	184 (50.4%)
**Highest educational level**	
Primary school	70 (19.2%)
Junior High school	31 (8.5%)
Secondary/Vocational school	139 (38.1%)
Tertiary	125 (34.2%)
BMI (kg/m²)*	28.7 (5.9)
**BMI category**	
Underweight	9 (2.5%)
Normal	81 (22.4%)
Overweight	143 (39.6%)
Obese	128 (35.5%)
Current systolic BP (mmHg)*	135.1 (9.1)
Current diastolic BP (mmHg)*	85.5 (11.8)
Quality of Life Score*	63.1 (6.6)
Knowledge score on Hypertension*	51.3 (25.7
MMAS score*	5.0 (1.6)
**Family APGAR score**	6.7 (2.2)
**Access to healthcare**	
With difficulty	111 (30.4%)
Easily	254 (69.6%)
**Healthcare quality satisfaction**	
Dissatisfied	232 (63.9%)
Satisfied	131 (36.1%)
**Dietary habits**	
Poor	30 (8.2%)
Fair	257 (70.4%)
Good	78 (21.4%)
**Physical activity**	
Never/Rarely	56 (15.3%)
Frequent	309 (84.7%)

* Mean (SD)

### Family functioning, quality of life, and medication adherence

Across the family functioning categories, medication adherence scores and quality of life score variations were assessed as shown in Table 2. Patients from highly functional (4.7 ± 1.8) had significantly higher medication adherence scores compared to those from moderately dysfunctional families and severely dysfunctional families (5.3 ± 1.3) (p = 0.034). Similarly, QoL scores were different across family functioning categories, with means of 61.4 ± 4.5, 64.6 ± 6.0, and 61.8 ± 6.9 for severely dysfunctional families, moderately dysfunctional families, and highly functional families, respectively (p < 0.001). Blood pressure control also showed significant differences across family functioning categories. Current systolic blood pressure was higher in patients from highly functional families (136.7 ± 9.6 mmHg) compared to those from moderately dysfunctional families (133.5 ± 8.4 mmHg) (p = 0.003). No significant difference was observed for diastolic blood pressure across the groups (p = 0.300).

**Table 2:** Blood Pressures, Quality of life and medication adherence by family functioning categories.

Characteristic	Severely Dysfunctional Family n = 16	Moderately Dysfunctional Family n = 173	Highly Functional Family n = 176	p-value
QoL score*	61.4 (4.5)	64.6 (6.0)	61.8 (6.9)	<0.001
MMAS score*	5.3 (1.3)	5.2 (1.4)	4.7 (1.8)	0.034
Current systolic BP (mmHg)*	134.1 (9.4)	133.5 (8.4)	136.7 (9.6)	0.003
Current diastolic BP (mmHg)*	90.4 (12.0)	85.4 (11.5)	85.2 (12.1)	0.3

*Mean (SD)

**Fig 1:**
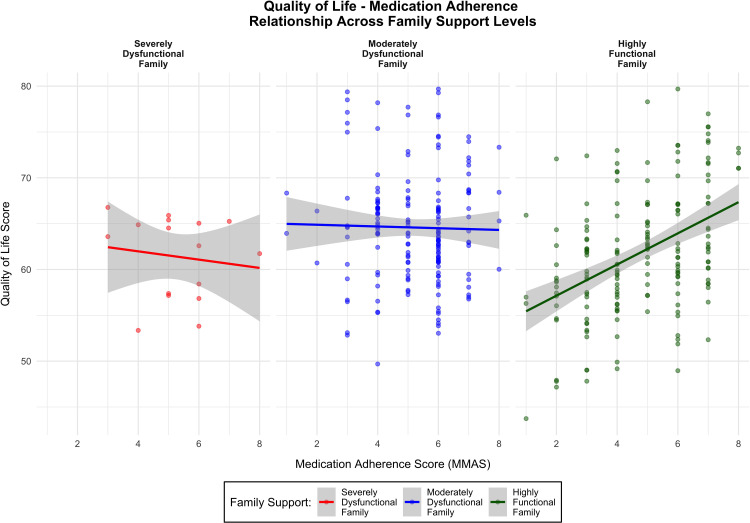
Scatter Plot of QoL and Medication Adherence across Family Support Levels.

A regression line on the scatter plot of medication adherence and QoL scores across family support levels revealed a positive association between medication adherence and QoL was strongest among patients from highly functional families, as indicated by the upward slope of the regression line in this group compared to those from moderately or severely dysfunctional families (Fig 1).

Among participants with highly functional families (Table 3), a unit increase in medication adherence score was strongly associated with a 1.70 higher QoL score (β = 1.70, 95% CI: 1.17–2.23, p < 0.001). An interaction model revealed a significant interaction between medication adherence and family support on QoL (β = 0.44, 95% CI: 0.26–0.63, p < 0.001),. Stratified analyses demonstrated that this relationship varied markedly by family functionality level.

**Table 3:** Linerar Regression Analysis of Quality of Life by Medication Adherence and Family Support.

	Interaction Model (All Groups)			Severely Dysfunctional			Moderately Dysfunctional			Highly Functional		
Characteristic	Beta(β)	95% CI	p-value	Beta(β)	95% CI	p-value	Beta(β)	95% CI	p-value	Beta(β)	95% CI	p-value
MMAS * Family APGAR score	0.44	0.26, 0.63	<0.001	−0.45	−2.37, 1.46	0.6	−0.09	−0.76, 0.57	0.8	1.70	1.17, 2.23	<0.001
R²	0.142			0.018			0.000			0.188		
Adjusted R²	0.135			−0.052			−0.005			0.183		
No. Obs.	365			16			173			176		
AIC	2,361			98.4			1,118			1,147		
BIC	2,381			101			1,128			1,157		

CI = Confidence Interval.

### Family support as a mediator between medication adherence and quality of life

The study further explored the role of family support in the medication adherence and QoL among. Table 4 presents the results of regression analyses for the mediator model and outcome model across five adjustment models. In the fully adjusted mediator model (Model 5), medication adherence was significantly associated with family support (β = −0.13, 95% CI: −0.25, −0.01). In the outcome model, both medication adherence (β = 0.87, 95% CI: 0.48, 1.3) and family support (β = −0.65, 95% CI: −0.99, −0.30) were significantly associated with QoL. The direct effect of medication adherence on QoL remained significant across all models, with the fully adjusted model showing an ADE of 0.87 (95% CI: 0.42, 1.26). The total effect of medication adherence on QoL in the fully adjusted model was 0.95 (95% CI: 0.51, 1.35). The mediation analysis further revealed that family support significantly mediated the relationship between medication adherence and QoL in the unadjusted model (ACME = 0.09, 95% CI: 0.02, 0.19), accounting for 8% of the total effect. This mediation effect persisted in the fully adjusted model (ACME = 0.08, 95% CI: 0.004, 0.18), with family support mediating 9% of the total effect. Similary, the mediation effect of family support persisted across the physical and mental component summary of QoL. It mediated 4-12% and 5-9% of the total effect for each component (see S1and S2 Tables) respectively across all the models.

**Table 4:** Linear Regression analysis Assessing adherence score relationship with family support (Mediator Model) and quality of life (Outcome Model).

	Model 1			Model 2			Model 3			Model 4			Model 5		
	β	95% CI	P-Value	β	95% CI	P-Value	β	95% CI	P-Value	β	95% CI	P-Value	β	95% CI	P-Value
**Mediator Model**															
MMAS score	−0.21	−0.35, −0.06	0.006	−0.15	−0.29, −0.01	0.041	−0.21	−0.35, −0.08	0.002	−0.13	−0.25, −0.01	0.037	−0.13	−0.25, −0.01	0.029
**Outcome Model**															
MMAS score	0.98	0.57, 1.4	<0.001	1.1	0.73, 1.5	<0.001	0.92	0.54, 1.3	<0.001	0.90	0.51, 1.3	<0.001	0.87	0.48, 1.3	<0.001
Family APGAR score	−0.42	−0.72, −0.13	0.005	−0.36	−0.65, −0.07	0.016	−0.65	−0.95, −0.36	<0.001	−0.56	−0.90, −0.23	0.001	−0.65	−0.99, −0.30	<0.001
**Mediation Effect Estimate**															
ACME	0.09	0.02, 0.19	0.008	0.05	−0.002, 0.13	0.076	0.14	0.04, 0.27	0.006	0.07	0.003, 0.17	0.038	0.08	0.004, 0.18	0.038
ADE	0.98	0.52, 1.42	<0.001	1.13	0.7, 1.53	<0.001	0.92	0.55, 1.32	<0.001	0.90	0.48, 1.28	<0.001	0.87	0.42, 1.26	<0.001
Total Effect	1.07	0.63, 1.50	<0.001	1.18	0.76, 1.58	<0.001	1.06	0.69, 1.47	<0.001	0.97	0.56, 1.36	<0.001	0.95	0.51, 1.35	<0.001
Proportion Mediated	0.08	0.02, 0.20	0.076	0.04	−0.001, 0.12	0.076	0.13	0.04, 0.28	0.006	0.07	0.003, 0.20	0.038	0.09	0.004, 0.23	0.038

*CI: Confiddence Interval*

ACME: Average Causal Mediation Effect

ADE: Average Direct Effect

*Model 1: Unadjusted*

*Model 2: Model 1 adjusted for age group, gender, and education level*

*Model 3: Model 2 + knowledge score on hypertension*

*Model 4: Model 3 + access to healthcare and healthcare quality satisfaction*

*Model 5: Model 4 + dietary habits and physical activity*

## Discussion

This study examined the mediating role of family support in the relationship between medication adherence and QoL among patients with hypertension receiving health services at the KNUST Hospital in Kumasi. In the mediator and outcome models (Table 4;S1-S2 Tables), Family APGAR showed negative associations with MMAS-8 and QoL. The mediation analysis of the overall QoL scores revealled that family support partially mediates this relationship, accounting for approximately 8–9% of the total effect. Similarly, mediation analysis of the physical and mental components of Qol revealled that family support partially accounted for 4–12% and 5–9% respectively of the total effect. Accordingly, the observed positive indirect effect results from the product of negative component paths, consistent with suppression. Family support demonstrates a suppressive indirect association between medication adherence and quality of life in this cross-sectional study. This complex pattern is further evidenced by the paradoxical finding that patients from moderately dysfunctional families reported higher QoL and medication adherence compared to those from highly functional families, who exhibited significantly higher systolic blood pressure and the lowest adherence scores. Such results suggest that highly functional family dynamics may inadvertently introduce overprotective stress or miscarried help that worsens health outcomes [18]. These findings further suggests that QoL and disease management in long-term care is affected other myriad of factors such as gender, knowledge gaps, educational status, and clinical factors like obesity and blood pressure control as shown in literature [19–21]. In the Ghanaian context, the family system acts as a central role in healthcare decision-making and disease management, where family members actively participate in treatment decisions and provide both emotional and physical support to patients with chronic conditions [22,23]. This cultural norm makes family support particularly relevant for hypertension management, as patients often rely on family members for medication reminders, transportation to healthcare facilities, dietary modifications, and emotional encouragement during treatment [22]. This complex pattern requires longitudinal confirmation before inferring causality or designing family-centered interventions.

The study further identified that individual association revealed an inverse relationship between the family APGAR score and medication adherence score as well as between family APGAR score and QoL scores in the regression models. The study observed similarities in the average QoL across the level of family functional groups although they were statistically different. However, considering QoL, family APGAR scores and medication adherence at the same time revealed significant differences across the family functional groups. A positive upward association between medication adherence and QoL was noted among patients from highly functional families. The observed differences in the QoL and medication adherence across family functioning categories further support the influence of family dynamics in hypertension management. This finding that family support significantly affect the relationship between medication adherence and QoL extends the existing evidence available in literature [9–11]. Also, the positive association between the interation effect of medication adherence and family support in relation to increase in QoL in this study aligns with other previous studies [11,24]. Family support may enhance QoL indirectly by providing emotional comfort, reducing stress, and fostering a sense of belonging [10]. Within the context of a supportive family, adherence to antihypertensive medications helps control blood pressure, reduce symptoms, and prevent complications, thereby improving physical well-being [11]. Additionally, the psychological benefits of adhering to prescribed treatments, including reduced anxiety about potential complications and greater perceived control over one’s health, may contribute to enhanced QoL [9,11]. Concurrently, it may facilitate medication adherence through practical assistance such as medication reminders, transportation to healthcare facilities, emotional encouragement, and reinforcement of healthy behaviours and knowledge on hypertension [10,11,24]. Higher knowledge about hypertension may facilitate better self-management, more effective communication with healthcare providers, and greater ability to navigate the healthcare system. The observed differences in the regression line and analysis of QoL and medication adherence across family functioning categories further supports the importance of family dynamics in hypertension management.

### Implications for clinical practice and policy

The findings of this study have some implications for clinical practice and policy. First, healthcare providers should assess family functioning and support systems as part of routine care for patients with hypertension. Identifying patients with limited family support may help target interventions to improve medication adherence and QoL. Second, involving family members in patient education and management plans could enhance treatment outcomes. Family-centered interventions that address knowledge gaps, communication skills, and practical support strategies may be particularly effective. At the policy level, initiatives to strengthen family support systems, such as caregiver education programs and support groups, should be integrated into hypertension management programs.

### Study strengths and limitations

This study has some strengths, including its use of validated instruments to measure key variables, comprehensive assessment of potential confounders, and application of robust mediation analysis techniques. However, some limitations should be acknowledged. The cross-sectional design precludes causal inferences about the relationships between medication adherence, family support, and QoL. Additionally, self-reported measures of medication adherence and family support may be subject to recall bias and social desirability effects. Also, the sample was drawn from a single hospital in an urban setting, potentially limiting generalizability to rural populations or other healthcare contexts in Ghana. Furthermore, while adjustments were made for several potential confounders, unmeasured factors may have influenced the observed relationships. Also, the study acknowleges the paradoxical effects of family functioning which may suggest suppression rather than mediation as seen in this analysis. Given the cross-sectional and simultaneous measurements, assumptions required for causal mediation (sequential ignorability and correct temporal ordering) cannot be verified; the suppressive pattern observed here should be confirmed prospectively.

## Conclusion

Family support demonstrates a suppressive indirect association between medication adherence and quality of life in this cross-sectional sample. These relationships persist after adjusting for sociodemographic characteristics, hypertension knowledge, healthcare access, and lifestyle factors. The findings highlight the importance of considering family dynamics in hypertension management and suggest that family-centered interventions may improve patient outcomes. This complex pattern requires longitudinal confirmation before inferring causality or designing family-centered interventions.

## Supporting information

S1 Table Linear Regression analysis Assessing adherence score relationship with family support (Mediator Model) and Physical Component Summary of quality of life (Outcome Model). (DOCX)

S2 Table Linear Regression analysis Assessing adherence score relationship with family support (Mediator Model) and Mental Component Summary of quality of life (Outcome Model). (DOCX)

## Abbreviations

ACME: Average Causal Mediation Effect
ADE: Average Direct Effect
APGAR: Adaptation, Partnership, Growth, Affection, Resolve
BMI: Body Mass Index
BP: Blood Pressure
CHRPE: Committee on Human Research, Publication and Ethics
CI: Confidence Interval
KNUST: Kwame Nkrumah University of Science and Technology
LMICs: Low- and Middle-Income Countries
MMAS: Morisky Medication Adherence Scale
OPD: Outpatient Department
QoL: Quality of Life
SD: Standard Deviation
SF-36: Short Form-36
SSA: Sub-Saharan Africa
