# Supplementary material for: Family support mediates the relationship between medication adherence and quality of life among patients with hypertension in Ghana: A cross-sectional study
Source: PLoS One. 2026 Jun 24;21(6):e0351632. doi: 10.1371/journal.pone.0351632 (PMC13293409; doi:10.1371/journal.pone.0351632)
Supplement: S1 Table — (DOCX) [file pone.0351632.s001.docx]

**S1 Table: Linear Regression analysis Assessing adherence score relationship with family support (Mediator Model) and Physical Component Summary of quality of life (Outcome Model)**

|  | | | |  | **Model 1** | | |  | **Model 2** | | |  | **Model 3** | | |  | **Model 4** | | |  | **Model 5** | | | | |
| --- | --- | --- | --- | --- | --- | --- | --- | --- | --- | --- | --- | --- | --- | --- | --- | --- | --- | --- | --- | --- | --- | --- | --- | --- | --- |
|  | | | |  | β | **95% CI** | **P-Value** |  | β | **95% CI** | **P-Value** |  | β | **95% CI** | **P-Value** |  | β | **95% CI** | **P-Value** |  | β | **95% CI** | | **P-Value** | |
| **Mediator Model** | | | |  |  |  |  |  |  |  |  |  |  |  |  |  |  |  |  |  |  |  | |  | |
| MMAS score | | | |  | -0.21 | -0.35, -0.06 | 0.006 |  | -0.15 | -0.29, -0.01 | 0.041 |  | -0.21 | -0.35, -0.08 | 0.002 |  | -0.13 | -0.25, -0.01 | 0.037 |  | -0.13 | -0.25, -0.01 | | 0.029 | |
|  | | | |  |  |  |  |  |  |  |  |  |  |  |  |  |  |  |  |  |  |  | |  | |
| **Outcome Model** | | | |  |  |  |  |  |  |  |  |  |  |  |  |  |  |  |  |  |  |  | |  | |
| MMAS score | | | |  | 0.86 | 0.39, 1.3 | <0.001 |  | 0.97 | 0.51, 1.4 | <0.001 |  | 0.80 | 0.34, 1.3 | <0.001 |  | 0.80 | 0.34, 1.3 | <0.001 |  | 0.79 | 0.32, 1.3 | | <0.001 | |
| Family APGAR score | | | |  | -0.36 | -0.69, -0.03 | 0.033 |  | -0.26 | -0.59, -0.08 | 0.13 |  | -0.50 | -0.85, -0.15 | 0.005 |  | -0.53 | -0.93, -0.13 | 0.009 |  | -0.56 | -0.97, -0.14 | | 0.008 | |
|  | | | |  |  |  |  |  |  |  |  |  |  |  |  |  |  |  |  |  |  |  | |  | |
| **Mediation Effect Estimate** | | | |  |  |  |  |  |  |  |  |  |  |  |  |  |  |  |  |  |  |  | |  | |
| ACME | | | |  | 0.07 | 0.004, 0.17 | 0.032 |  | 0.04 | -0.01, 0.12 | 0.152 |  | 0.11 | 0.02, 0.24 | 0.012 |  | 0.07 | 0.001, 0.18 | 0.048 |  | 0.07 | 0.003, 0.17 | | 0.036 | |
| ADE | | | |  | 0.85 | 0.38, 1.35 | <0.001 |  | 0.97 | 0.48, 1.42 | 0.002 |  | 0.80 | 0.30, 1.24 | 0.006 |  | 0.80 | 0.30, 1.21 | <0.001 |  | 0.79 | 0.30, 1.28 | | 0.004 | |
| Total Effect | | | |  | 0.93 | 0.47, 1.41 | <0.001 |  | 1.01 | 0.52, 1.45 | <0.001 |  | 0.91 | 0.42, 1.35 | <0.001 |  | 0.87 | 0.39, 1.29 | <0.001 |  | 0.85 | 0.37, 1.37 | | 0.002 | |
| Proportion Mediated | | | |  | 0.08 | 0.01, 0.22 | 0.032 |  | 0.04 | -0.01, 0.14 | 0.152 |  | 0.12 | 0.02, 0.34 | 0.012 |  | 0.07 | 0.001, 0.29 | 0.048 |  | 0.09 | 0.003, 0.26 | | 0.038 | |
|  |  |  |  | *CI: Confiddence Interval*  ACME: Average Causal Mediation Effect  ADE: Average Direct Effect  *Model 1: Unadjusted*  *Model 2: Model 1 adjusted for age group, gender, and education level*  *Model 3: Model 2 + knowledge score on hypertension*  *Model 4: Model 3 + access to healthcare and healthcare quality satisfaction*  *Model 5: Model 4 + dietary habits and physical activity* | | | | | | | | | | | | | | | | | | |  | |  |
