# Supplementary material for: Family support mediates the relationship between medication adherence and quality of life among patients with hypertension in Ghana: A cross-sectional study
Source: PLoS One. 2026 Jun 24;21(6):e0351632. doi: 10.1371/journal.pone.0351632 (PMC13293409; doi:10.1371/journal.pone.0351632)
Supplement: S2 Table — (DOCX) [file pone.0351632.s002.docx]

**S2 Table: Linear Regression analysis Assessing adherence score relationship with family support (Mediator Model) and Mental Component Summary of quality of life (Outcome Model)**

|  | | | |  | **Model 1** | | |  | **Model 2** | | |  | **Model 3** | | |  | **Model 4** | | |  | **Model 5** | | | | |
| --- | --- | --- | --- | --- | --- | --- | --- | --- | --- | --- | --- | --- | --- | --- | --- | --- | --- | --- | --- | --- | --- | --- | --- | --- | --- |
|  | | | |  | β | **95% CI** | **P-Value** |  | β | **95% CI** | **P-Value** |  | β | **95% CI** | **P-Value** |  | β | **95% CI** | **P-Value** |  | β | **95% CI** | | **P-Value** | |
| **Mediator Model** | | | |  |  |  |  |  |  |  |  |  |  |  |  |  |  |  |  |  |  |  | |  | |
| MMAS score | | | |  | -0.21 | -0.35, -0.06 | 0.006 |  | -0.15 | -0.29, -0.01 | 0.041 |  | -0.21 | -0.35, -0.08 | 0.002 |  | -0.13 | -0.25, -0.01 | 0.037 |  | -0.13 | -0.25, -0.01 | | 0.029 | |
|  | | | |  |  |  |  |  |  |  |  |  |  |  |  |  |  |  |  |  |  |  | |  | |
| **Outcome Model** | | | |  |  |  |  |  |  |  |  |  |  |  |  |  |  |  |  |  |  |  | |  | |
| MMAS score | | | |  | 1.1 | 0.62, 1.6 | <0.001 |  | 1.3 | 0.81, 1.8 | <0.001 |  | 1.00 | 0.58, 1.5 | <0.001 |  | 1.00 | 0.53, 1.5 | <0.001 |  | 0.95 | 0.49, 1.4 | | <0.001 | |
| Family APGAR score | | | |  | -0.49 | -0.84, -0.14 | 0.006 |  | -0.45 | -0.80, -0.10 | 0.011 |  | -0.65 | -1.20, -0.44 | <0.001 |  | -0.59 | -1.00, -0.19 | 0.004 |  | -0.73 | -1.10, -0.33 | | <0.001 | |
|  | | | |  |  |  |  |  |  |  |  |  |  |  |  |  |  |  |  |  |  |  | |  | |
| **Mediation Effect Estimate** | | | |  |  |  |  |  |  |  |  |  |  |  |  |  |  |  |  |  |  |  | |  | |
| ACME | | | |  | 0.10 | 0.02, 0.23 | 0.008 |  | 0.07 | -0.001, 0.17 | 0.046 |  | 0.17 | 0.06, 0.31 | <0.001 |  | 0.08 | 0.001, 0.18 | 0.038 |  | 0.10 | 0.01, 0.20 | | 0.032 | |
| ADE | | | |  | 1.11 | 0.60, 1.59 | <0.001 |  | 1.29 | 0.81, 1.75 | <0.001 |  | 1.04 | 0.56, 1.49 | <0.001 |  | 1.00 | 0.52, 1.42 | <0.001 |  | 0.95 | 0.48, 1.44 | | 0.002 | |
| Total Effect | | | |  | 1.21 | 0.70, 1.69 | <0.001 |  | 1.35 | 0.87, 1.82 | <0.001 |  | 1.22 | 0.72, 1.69 | <0.001 |  | 1.08 | 0.58, 1.52 | <0.001 |  | 1.05 | 0.54, 1.54 | | <0.001 | |
| Proportion Mediated | | | |  | 0.08 | 0.01, 0.20 | 0.008 |  | 0.05 | -0.001, 0.13 | 0.046 |  | 0.14 | 0.05, 0.28 | <0.001 |  | 0.07 | 0.001, 0.18 | 0.038 |  | 0.09 | 0.01, 0.22 | | 0.032 | |
|  |  |  |  | *CI: Confiddence Interval*  ACME: Average Causal Mediation Effect  ADE: Average Direct Effect  *Model 1: Unadjusted*  *Model 2: Model 1 adjusted for age group, gender, and education level*  *Model 3: Model 2 + knowledge score on hypertension*  *Model 4: Model 3 + access to healthcare and healthcare quality satisfaction*  *Model 5: Model 4 + dietary habits and physical activity* | | | | | | | | | | | | | | | | | | |  | |  |
